# The Challenges of Arterial Hypertension

**DOI:** 10.3389/fcvm.2015.00002

**Published:** 2015-02-03

**Authors:** Gian Paolo Rossi

**Affiliations:** ^1^Department of Medicine (DIMED), Internal Medicine 4, University of Padua, Padua, Italy

**Keywords:** aldosterone, hyperaldosteronism, heart damage, cardiac dysfunction

## Introduction

The blood pressure (BP) is the force generated by the heart that drives circulation of the blood. After Scipione Riva-Rocci first introduced the sphygmomanometer at the beginning of last century, for decades arterial hypertension (hypertension) was considered to be “essential” for securing the perfusion of vital organs, as the brain, heart, and kidney. The term “essentielle hypertonie,” coined by the German clinicians, was indeed meant to underline the fact that BP, alongside the heart beat, is the essence of life, as there can be no life without BP. This led to contend that the higher the BP values, the better for vital organs perfusion, a view testified by Paul Dudley White’s, one of the top cardiologists of the last century, statement: “*Hypertension may be an important compensatory mechanism, which should not be tampered with, even were it certain we could control it*.”

It took the randomized clinical trials in the eighties of the last century to dismantle this misconception, and finally prove that lowering BP was beneficial, e.g., better than placebo for lowering cardiovascular events (1–3). Hypertensiologists should indeed be proud of the fact that antihypertensive treatment has been the first cardiovascular therapy whose efficacy has been proven beyond doubts with randomized clinical trials and use of hard end points. Several decades of epidemiological and experimental research, along with a number of further large-scale clinical trials [reviewed in Ref. (4–6)], have now led the widely accepted concept that hypertension is the “number one” cardiovascular risk factor, not only because of its very high prevalence but also for its tight relationship with cerebro- and cardiovascular events.

In westernized countries around 40% of the adult men and women have hypertension, which puts them at an excess risk of stroke, both hemorrhagic and ischemic, acute coronary syndromes, heart failure, and chronic kidney disease with the ensuing need for renal replacement therapy. Considering that worldwide about 13.5% of premature deaths were attributed to hypertension in 2001, the World Health Organization defined hypertension “*a humanitarian tragedy on a planetary scale*” (7, 8).

## Achievements in the Management of Hypertension

The increasing awareness of the detrimental effects of hypertension has been paralleled by the development of an overwhelming amount of knowledge on cardiovascular pathophysiology. This has had enormous fallout in the diagnostic approach and treatment of many other cardiovascular diseases. It has also led to the development of effective non-pharmacologic measures and pharmacologic agents. Accordingly, current guidelines recommend a number of life-style changes to improve the control of BP values, both in primary prevention and as a valuable addition to pharmacologic treatment. The latter has made terrific steps since the Kempner fruits and rice diet, and the first introduction of the ganglio-plegic drugs in the sixties of the previous century. Accordingly, more than 100 drugs, alone or in predefined combinations, are now available for the treatment of hypertension. Notwithstanding these progresses, far too many hypertensive patients continue to have BP far above the target values that would be desirable based on their overall cardiovascular risk profile. Current, estimates of uncontrolled hypertension indicate that up to 30–40% of the patients have BP values above target. Hence, there are several challenges that need to be addressed in the near future (Table 1).

**Table 1:** **The challenges of arterial hypertension**.

∙ To develop more effective and widely applicable strategies for improving BP control worldwide
∙ To develop more aggressive strategies to diagnose and treat RH
∙ To integrate the top-down guidelines approach with diffuse postgraduate training programmes in hypertension
∙ To increase the awareness and detection of secondary forms of hypertension
∙ To facilitate the detection, diagnosis, and treatment of primary aldosteronism
∙ To improve the detection and treatment of renovascular hypertension
∙ To investigate the feasibility of a genomic-based approach for individualizing the management of arterial hypertension

## Problem of Resistant Hypertension

Considering that uncontrolled hypertension leads to excess target organ damage and complications (9), the patients with resistant hypertension (RH) are still exposed to an excess risk of CV events (10). Recognizing the importance of this re-emerging problem the major scientific societies, both in Europe and in North America, have introduced the concept of drug “resistant” or refractory hypertension and developed definitions for this condition (Table 2). Albeit varying slightly across societies, these definitions are useful in drawing attention to this problem. They also underline the fact that hypertension is a heterogeneous condition whose pathophysiological mechanisms still remain elusive in many patients.

**Table 2:** **Definitions of resistant hypertension according to major scientific societies**.

Society/Year/Reference	Definition
JNC 7 (2003)	Failure to reach BP goal in patients who are adhering to full doses of **an appropriate 3-drug regimen that includes a diuretic**
AHA Scientific Statement 2008	“… blood pressure that remains above goal despite the concurrent use of 3 antihypertensive agents of different classes. **Ideally, one of the 3 agents should be a diuretic**.” It includes patients whose blood pressure is controlled but require 4 or more medications to do so
ESH/ESC Guidelines 2007	BP ≥140/90 mmHg despite treatment with at least 3 drugs (including a diuretic) in adequate doses and after exclusion of spurious hypertension such as isolated systolic hypertension and failure to use large cuffs on large arms
BHS (2011)	Someone whose BP is not controlled to <140/90 mmHg despite optimal or best-tolerated doses of third line treatment
ESH/ESC Guidelines 2013 (15)	“… when a therapeutic … strategy that includes appropriate life-style measures plus a diuretic and two other antihypertensive drugs belonging to different classes at adequate doses (but not necessarily including a mineralocorticoid receptor antagonist) fails to lower SBP and DBP values to 140 and 90 mmHg, respectively”

*JNC, Joint National Committee; AHA, American Heart Association; ESH, European Society of Hypertension; ESC European Society of Cardiology; BHS, British Hypertension Society. Bold indicates the importance of diuretics in the definition*.

Hence, a major challenge for the coming years entails developing more aggressive research and novel therapeutic strategies for RH, if we are not going to passively accept a situation that takes still too many lives every year worldwide.

## Current Strategies to Fight Hypertension

The general approach adopted thus far by most international scientific societies and national societies to address the issue of improving the control of hypertension, has been through the release of guidelines for the management of hypertension. For example, the European Society of Cardiology (ESC) and the European Society of Hypertension (ESH) have jointly released guidelines repeatedly in the last two decades (11–15) and more recently in 2013.

These guidelines are commendable in that they provided useful practical information on evaluation of biomarkers for risk stratification, life-style measures, and strategy of management, including use of the most recent sympathetic renal denervation and baroreceptor stimulation therapy. However, in spite of their publication in multiple journals, and notwithstanding these progresses in treatment, the prevalence of uncontrolled hypertension did not fall, but rather continued to rise. This is clear-cut evidence that guidelines – based approaches are by themselves not sufficient to solve the problem of the poor BP control at the population level. Looking at the latest edition of the ESH/ESC guidelines some reasons for this failure are readily evident. The paper is made of 76 pages, 14 tables, 5 figures, and 735 references (15). This is an overwhelming amount of information that exceeds by far the amount evidence that any busy general practitioner or practicing clinicians dealing with a multitude of patients crowding the waiting rooms of their outpatient clinics can ever possibly read. Recognizing this problem the ESH has prepared a digest version of the European guidelines, but the impact of this “simplified” approach is still to be seen.

As common to other guidelines, the method exploited in writing these guidelines was a “top-down” approach, e.g., from level of evidence and class of recommendation, mainly produced by randomized clinical trials, to practical advices, rather than a problem-oriented “bottom-up” approach. While understandable and obviously scientifically sound, this top-down approach contributed to building up the complexity of the guidelines, and to produce their resulting scant impact in clinical practice.

Hence, a major challenge facing the hypertension community is to conceive and develop novel strategies for continuous medical education in the field of hypertension, having always in mind the final users, e.g., the doctors and the patients.

Additionally, given the complexity of the hypertension field, which requires a multidisciplinary medical formation, with notions spanning from cardiology, to endocrinology, to internal medicine, to nephrology to clinical pharmacology, at least in Europe, there is an urgent need for developing postgraduate training programs in hypertension. To the best of my knowledge the only such programs is the International PhD Program in Arterial Hypertension and Vascular Biology, which was developed at our University in 2001 in conjunction with the Medical Academy of Gdansk and with Charitè University in Berlin and is to be extended to the University of Maastricht next year.

## Under Diagnosis and Under Treatment of Secondary Hypertension

Undiagnosed forms of secondary hypertension likely contribute substantively to the on-going epidemics of uncontrolled hypertension. Currently, at referral centers for hypertension, up to 25% of the patients are found to be affected by secondary forms of hypertension, a figure 5- to 10-fold higher than those reported in the major textbooks. The latter reported data mainly based on dated epidemiologic studies that mostly made use of obsolete diagnostic strategies. This apparent increase in prevalence of secondary hypertension means that if a proper diagnostic workup were systematically exploited, an underlying cause of hypertension could be identified in about one patient every four. Hence, a substantial proportion of the patients now mislabeled as “essential hypertension” can thus be either long-term cured or, at least, their BP values can be brought under control, as recently shown to be the case for primary aldosteronism (16).

In spite of the high rate of secondary hypertension, in the most recent ESC/ESH guidelines only a quarter page was devoted to the screening and diagnostic workup of these forms (15), which left practicing physicians with the perception that this is a “niche” problem, while it is altogether evident that it is not. Of little help in this regard was the release of specific guidelines for some endocrine forms of secondary hypertension, including primary aldosteronism (17) and pheochromocytoma (18). These guidelines were prepared by “specialists for specialists.” Therefore, they are perceived to be too complex to be effectively implemented into clinical practice. Accordingly, far too many potentially curable patients are left with a questionable diagnosis of primary (essential) hypertension simply because they were not properly investigated beforehand.

## The Problem of Primary Aldosteronism

There is no doubt that primary aldosteronism is the most common endocrine, and probably also the most under diagnosed and under treated, cause of hypertension. As a result of this, many patients, who could be long-term cured (16), are exposed to antihypertensive treatment for their entire lifetime. At the same time, they remain exposed to an excess risk of CV events. A number of reasons can account for this ominous situation. The still diffuse, albeit wrong, perception that primary aldosteronism is exceptionally rare is the first of them. The second entails the fact that, as already mentioned, the diagnostic workup is too complex (17). The development of simplified diagnostic algorithms based on novel, omics-derived biomarkers is therefore, a major challenge for the hypertension community for the years to come. These algorithms could then be used to screen these patients leading to a more targeted treatment with ensuing improved BP control.

## The Problem of Atherosclerotic Renovascular Hypertension

Atherosclerotic renal artery stenoses (ARAS) are increasingly found due to aging of the population. They account for about 90% of all renal artery lesions (19) and, through cholesterol emboli, they can lead to cortical infarcts, ischemic nephropathy, and eventually chronic renal failure. Moreover, through activation of the renin–angiotensin–aldosterone system (RAAS) they have other nefarious effects, including inflammation, oxidative stress, endothelial dysfunction, hypertension, thus accelerating widespread cardiovascular disease (20).

Although stenosis is generally held to attain hemodynamic significance only when the luminal narrowing is at least 70%, as compared to the nearby unaffected vessel or, if between 50 and 70%, when the trans-stenotic peak or mean pressure gradient is >20 or >10 mmHg, respectively (21), these threshold values are to be regarded cautiously as discussed elsewhere (22).

Until 1978, when Grüntzig introduced percutaneous transluminal renal angioplasty (PTRA) (23), patients with ARAS were treated medically or surgically. Observational, retrospective, and controlled trials thereafter suggested that PTRA could be beneficial over medical treatment for preserving renal function and improving BP control if followed by stenting [reviewed in Ref. (22)]. However, PTRA plus stenting (PTRAS) could not unequivocally be shown to overcome medical therapy in the few available randomized clinical trials. These trials, however, had limitations in study design, inclusion and exclusion criteria, and treatment options that led to seriously challenge their results and conclusions (24–26).

A recent meta-analysis of seven studies comprising a total of 2155 patients, including the recently reported CORAL Study, showed that compared to baseline, diastolic BP fell more at follow-up in patients in the endovascular than in the medical treatment arm (*P* = 0.002) (22). Moreover, patients with ARAS receiving endovascular treatment required less anti-antihypertensive drugs at follow-up than those medically treated. Hence, the lower diastolic BP was achieved despite a greater reduction in the mean number of antihypertensive drugs (necessary to control BP (*P* < 0.001) (22). Identical conclusions were reached by at least three meta-analyses: the first on 527 patients recruited in five prospective multicentre FDA-approved trials (27), the second, a Cochrane meta-analysis, for both diastolic BP and lowered antihypertensive therapy (28), and the third for lowered antihypertensive therapy (29).

Conclusive evidence that PTRAS lowers incident cardiovascular and renal events more than medical therapy in patients with ARAS remains, however, to be provided, likely because all these studies were too small in size and too short in duration to achieve this result. Accordingly, the optimal treatment of ARAS patients remains a highly controversial issue. Currently, this translates in ample variation in clinical practice, even though available guidelines on treatment of ARAS identified some compelling indications to revascularization. These recommendations are based on class I, level of evidence B (Table 3) (30).

**Table 3:** **Questions that need to be answered in future trials in hypertension**.

∙ Does the lower BP the better the outcome apply to all conditions and organs?
∙ Are there long-term benefits, e.g., beyond the duration of the RCT?
∙ Are there BP-independent protective properties that differ among different classes of antihypertensive drugs?
∙ Is there a protective effect of lowering BP in acute stroke?
∙ Does antihypertensive treatment prevent cognitive function decay/dementia?
∙ Is there a beneficial effect of early treatment in delaying target organ damage/reduce residual risk?
∙ Does BP variability represent a target for treatment beyond absolute BP values?
∙ Are there beneficial effects in mild hypertensives, who are at low-intermediate risk?
∙ What are the real beneficial effects of life-style changes in reducing long-term risk?
∙ What is the clinical efficacy of the gene-based prescribing strategy?
∙ Does the increment in efficacy or safety overcome the cost of genetic testing?
∙ Will genetic stratification be useful in clinical trials to evaluate new drugs?
∙ Will genetic information be valuable for the design of new agents that are likely to produce less adverse effects in genetically susceptible patients?

Therefore, another major challenge is to plan well-designed clinical research to improve clinical care of the increasing number of elderly patients with atherosclerotic renal artery stenosis and hypertension. The Medical and Endovascular Treatment of Atherosclerotic Renal Artery Stenosis (METRAS) now on-going (METRAS study)^1^ (31) can be an example of these.

## Gender-Related Differences in BP

There are clear-cut gender-specific differences that contribute to the well-known dimorphism in CV risk between genders: fertile women have lower BP values than age-matched men; the opposite is true after menopause. Notwithstanding this, the mechanisms underlying this BP gender dimorphism are just starting to be unraveled. For example, investigating the gender-related regulation of aldosterone production Caroccia et al recently found a major role for estradiol. The estrogen tonically inhibits aldosterone production by acting through beta-receptor, as when this effect is removed by pharmacological blockade or molecular knocking down of this nuclear receptor, the hormone exerts a potent stimulatory role on aldosterone production acting via the GEPR-1 receptor (32). Establishing whether such mechanisms is important for determining the changes in BP values in women with aging, during the menstrual cycle, or during long-term treatment with estrogen-receptor modulators, which are being prescribed to a multitude of women with estrogen-receptor positive breast cancer, is a key question to be addressed with specific research in the future decade.

## Lights and Shadows of Genomics of Hypertension

In the last two decades, impressive efforts and resources have been devoted to the search for the genes causing hypertension. Advancements have been accomplished for some monogenic forms, like Familial Hyperaldosteronism (FH)-1 (GRA), Liddle syndrome, Gordon syndrome, and more recently FH-3 (33–35). Almost invariably the rewarding strategy has been to start from phenotypically well-characterized pedigrees, mostly involving cases of endocrine hypertension, use linkage analysis to pinpoint the underlying genes and mutations. Results of large-scale association studies have not, however, been equally impressive and rewarding even with use of the more sophisticate and expensive genome-wide scanning studies. Even when association of BP with genetic variants were identified, the relative risk of hypertension in the individual carrying the variants was tiny. Consequently, the impact of these studies on clinical practice has been negligible.

A major challenge for the future is therefore to improve the stratification of the hypertensive patients not only for gender, age, and/or BP values but also for traits that can better lend themselves for the genetic analysis.

## Pharmacogenomics and Future Perspectives

Pharmacogenomics is attracting increasing attention in scientific circles and in the popular press, mainly because of the promise of personalized Medicine. Short of being able to cure or to prevent hypertension in the majority of the cases, genomics research might eventually fulfill every hypertensiologist’s dream, which is to move from the current “trial-and-error” drug treatment strategy to offering patients a precisely targeted drug at a precisely calibrated dose – and to do so without wreaking adverse effects.

While the vision of uncovering new targets for pharmacologic intervention and of creating novel agents for individualized treatment glimmers tantalizingly distantly on the horizon, much closer to application might be the use of information about how genetic variations affect the efficacy of drugs to guide prescribing decisions for agents currently on the market.

There are sound reasons to predict that pharmacogenomics will eventually succeed in fulfilling this is promise. Technological advances that enable to identify millions of DNA sequence variations swiftly and inexpensively, and to correlate them with phenotypic characteristics, are occurring with a restless rhythm (36). Indeed, it is conceivable that someday soon DNA sequencing will be a routine part of the workup for hypertensive patients, at the very least to identify a patient’s sensitivity to drugs that are likely to produce adverse effects.

The National Institutes of Health consortium on pharmacogenetics research network (PGRN) is collecting information on how genetic variation contributes to differences in drug response among patients and data about specific proteins, genes, and pathways is being integrated into the Pharmacogenetics and Pharmacogenomics Knowledge Base (37). Hence, we might not be a long way from the day when a patient presents a DNA “chip,” a key-chain tag bearing his electronic health record, to a physician and gets a dose of personalized treatment of hypertension.

However, a good deal of research still needs to be performed in clinical trails to address multiple questions (Table 3). Among such questions a major place regards the clinical trials to evaluate the clinical efficacy of the gene-based prescribing strategy, and to determine whether the increment in efficacy or safety warrants the cost of genetic testing. Moreover, genetic stratification in clinical trials will be needed to evaluate new drugs – and indeed, genetic information will be invaluable for the design of new agents to serve as alternatives to existing drugs that are likely to produce adverse effects in genetically susceptible patients.

## Conflict of Interest Statement

The author declares that the research was conducted in the absence of any commercial or financial relationships that could be construed as a potential conflict of interest.

## Footnotes

^1^http://clinicaltrials.gov/ct2/show/NCT01208714
